# Urticarial Vasculitis: A Case Report of Comorbid Generalized Anxiety Disorder

**DOI:** 10.7759/cureus.86802

**Published:** 2025-06-26

**Authors:** Katherine Rijo Florimon, Lisa A Bueno Fernandez, Nnenna B Emejuru, Akinahom Asressahegn, Shivani Saini

**Affiliations:** 1 Medicine, Pontificia Universidad Católica Madre y Maestra, Santiago de los Caballeros, DOM; 2 Psychiatry and Behavioral Sciences, College of Medicine, Imo State University, Orlu, NGA; 3 Medicine, St. Paul's Hospital Millennium Medical College, Charlotte, USA; 4 Clinical Psychology, Hurley Mental Health, Flint, USA

**Keywords:** chronic spontaneous urticaria, corticosteroid therapy, cutaneous small-vessel vasculitis, differential diagnosis, generalized anxiety disorder (gad), normocomplementemic urticarial vasculitis, psychological issues due to stress, sedentary behaviour, systemic symptoms, urticarial vasculitis

## Abstract

Urticarial vasculitis (UV) is a rare small-vessel vasculitis that can mimic chronic spontaneous urticaria but is distinguished by longer-lasting, painful lesions and potential systemic involvement. We present the case of a 29-year-old woman with untreated generalized anxiety disorder (GAD) who developed a painful, purpuric rash following a period of intense psychological stress and prolonged sedentary behavior while engaged in academic duties. Her symptoms included gastrointestinal discomfort, arthralgia, and myalgia. Laboratory investigations revealed normal inflammatory markers, normal complement levels, and negative autoimmune serologies, consistent with a diagnosis of normocomplementemic urticarial vasculitis (NUV). Antihistamines were ineffective; however, the patient responded well to oral corticosteroids. This case emphasizes the need to consider NUV in the differential diagnosis of persistent urticarial lesions with systemic symptoms and poor response to antihistamines. It also highlights the potential role of psychological stress as a trigger, particularly in individuals with comorbid psychiatric conditions such as GAD. A multidisciplinary approach integrating dermatologic, immunologic, and psychological evaluation is essential for optimal management.

## Introduction

Urticarial vasculitis (UV) is a type of small vessel vasculitis that occurs more frequently in women [1]. While it is most often idiopathic, it may also be associated with malignancies, autoimmune diseases, emotional stress, anxiety, alcohol consumption, physical exertion, and certain medications, including angiotensin-converting enzyme inhibitors (ACE inhibitors), penicillin, and nonsteroidal anti-inflammatory drugs (NSAIDs) [1,2]. The clinical presentation of UV is variable and may include persistent, indurated wheals, pruritus, fatigue, arthralgia, myalgia, gastrointestinal symptoms (nausea, vomiting, diarrhea), as well as pain or burning sensations of the skin [3]. Histopathologically, UV is characterized by leukocytoclastic vasculitis [2]. Corticosteroids are effective in managing over 80% of cases; however, antihistamines may also be used to relieve symptoms [2,3]. UV is broadly classified into two subtypes: hypocomplementemic urticarial vasculitis (HUV) and normocomplementemic urticarial vasculitis (NUV). NUV is more common and typically limited to the skin [3].

## Case presentation

**Figure 1:**
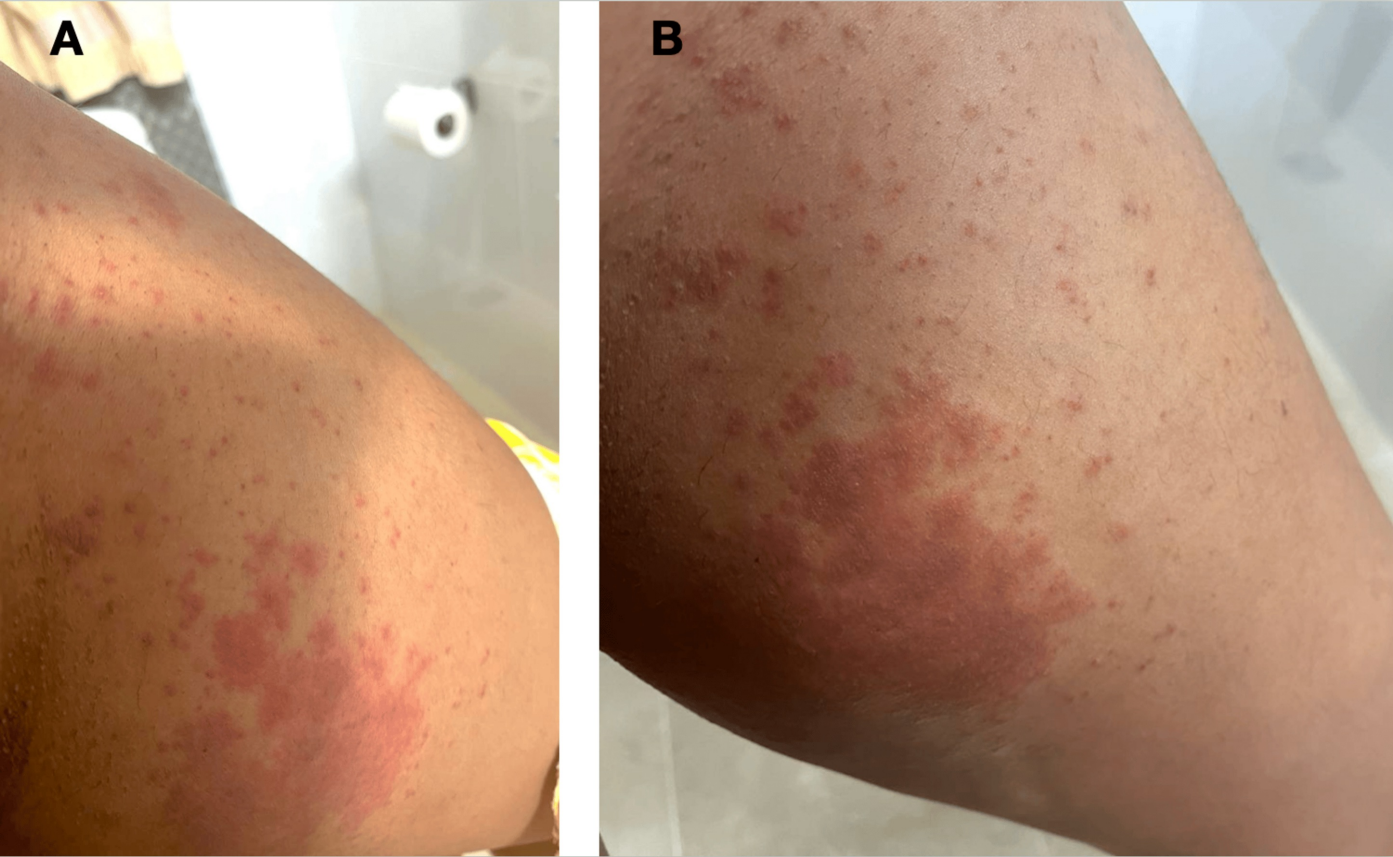
(A) Inner aspect of the left thigh on day 3 of presentation, showing lesions with progression to a reddish-purple discoloration (B) Inner thigh on day 4, demonstrating coalescence of the lesions into a larger, raised reddish-purple plaque with surrounding petechiae

A 29-year-old female graduate with a known history of generalized anxiety disorder (GAD) presented with a three-day history of a red, itchy rash localized to the left gluteal region, left inner thigh, and lower back (Figure 1). The rash developed following a prolonged period of psychological stress, academic pressure, generalized anxiety episodes, and increased sedentary behavior, with the patient reporting multiple hours spent seated at her computer for academic reasons.

On day one, the patient noticed the appearance of multiple small, reddish, pruritic spots over the left gluteal region. These subsequently coalesced into a larger, raised erythematous plaque with surrounding petechiae extending toward the lower back. By day three, the lesions had expanded to the inner thigh, deepened in color to a reddish-purplish hue, and involved a larger portion of the left lower back. The lesions became painful and were associated with a burning sensation. The patient had used antihistamines before the visit, but they did not provide symptomatic relief.

The patient also reported abdominal pain, diarrhea, arthralgia, and myalgia. She was not on any regular medications and had no significant past medical history aside from untreated GAD. There was no recent travel or known exposure to new medications, allergens, or infections. Dermatologic examination revealed a well-demarcated, elevated erythematous purpuric plaque with petechiae in adjacent areas, but no mucosal involvement.

While a skin biopsy was initially considered, the patient declined the procedure due to discomfort, lack of financial resources, and personal preference. Additionally, the case was managed in a resource-limited outpatient setting with constrained access to dermatopathology services. Clinical judgment played a key role in the decision not to pursue biopsy: the lesions exhibited features consistent with urticarial vasculitis, complement levels were normal, and the patient showed a rapid response to corticosteroids. After the treatment, the lesions were already improving significantly, without signs of progression or systemic deterioration. Consequently, a biopsy was deemed unlikely to alter management.

Laboratory investigations were largely within normal limits, including a complete blood count (CBC), metabolic panel, liver and renal function tests, and thyroid function tests (which revealed a slightly low free T4 of 0.89 ng/dL). Inflammatory markers, such as C-reactive protein (CRP), were also normal (<0.6 mg/L). Autoimmune serologies, including ANA, ANCA, anti-dsDNA, anti-MPO, anti-PR3, and rheumatoid factor, were negative. Complement levels (C3, C4) and immunoglobulin panels were within normal limits. Mild abnormalities included slight eosinophilia (5.1%, absolute 0.42 x10³/µL) and monocytosis (10.70%, absolute 0.88 x10³/µL). Laboratory testing results are summarized in Tables 1, 2, 3.

**Table 1:** Complete blood count with partial hepatic and renal panel and LDH RBC: red blood cell count; MCV: mean corpuscular volume; MCH: mean corpuscular hemoglobin; MCHC: mean corpuscular hemoglobin concentration; WBC: white blood cell count; AST: aspartate aminotransferase; ALT: alanine aminotransferase; BUN: blood urea nitrogen; LDH: lactate dehydrogenase

Test	Result	Reference Range
RBC	4.38	3.70–5.00 x10⁶/µL
Hemoglobin	13.3	12.0–16.0 g/dL
Hematocrit	39.2%	36.0–46.0%
MCV	89.5	80–99 fL
MCH	30.4	25.0–35.0 pg
MCHC	33.9	31.0–36.0 g/dL
WBC	8.20	4.0–10.00 x10³/µL
Neutrophils (#)	4.69	1.80-7.80 x10³/µL
Lymphocytes (#)	2.09	1.00–3.40 x10³/µL
Monocytes (#)	0.88	0.00–0.80 x10³/µL
Eosinophils (#)	0.42	0.00–0.40 x10³/µL
Basophils (#)	0.10	0.00–0.20 x10³/µL
Neutrophils	57.30%	40–75%
Lymphocytes	25.50%	16–46%
Monocytes	10.70%	0–12%
Eosinophils	5.10%	0–7%
Basophils	1.2%	0–2%
Platelets	272	150–400 x10³/µL
AST	17	0–32 U/L
ALT	15	0–33 U/L
BUN	14	6–20 mg/dL
LDH	170	135–214 U/L
Creatinine	0.91	0.50-0.90 mg/dL

**Table 2:** Other relevant laboratory results ESR: erythrocyte sedimentation rate; CRP: C-reactive protein; ANA: antinuclear antibody; ANCA: antineutrophil cytoplasmic antibody; Anti-MPO: anti-myeloperoxidase antibody; Anti-PR3: anti-proteinase 3 antibody; Anti-DNA: anti-double stranded DNA antibody; Anti-Thyroglobulin: antibody against thyroglobulin; Anti-TPO: anti-thyroid peroxidase antibody; TSH: thyroid stimulating hormone; T4 Free: free thyroxine; IgA: immunoglobulin A, IgM: immunoglobulin M; C3: complement 3; C4: complement 4

Test	Result	Reference Range
ESR	15	0–20 mm/hr
CRP	<0.6	≤5.0 mg/L
ANA	<1.80	<1.80 UI/mL – Negative
ANCA Screen	Negative	Negative
Anti-MPO	<3.2	<20 CU – Negative
Anti-PR3	<2.3	<20 CU – Negative
Anti-DNA	< 1:10	<1:10 CU – Negative
Anti-Thyroglobulin	14.90	0.00–115.00 UI/mL
Anti-TPO	18.10	0.00–34.00 UI/mL
TSH	2.94	0.27–4.20 µUI/mL
T4 Free	0.89	0.93–1.71 ng/dL
Rheumatoid Factor	< 10	0–14 U/mL
IgA	169	70-400 mg/L
IgM	178	40–230 mg/L
Complement C3	114	90–180 mg/dL
Complement C4	27	10–40 mg/dL

**Table 3:** Other relevant laboratory results Chlamydia IgA: immunoglobulin A antibody against Chlamydia; Cytomegalovirus IgM II: immunoglobulin M antibody against cytomegalovirus, type II assay; Epstein-Barr-VCA IgM: immunoglobulin M antibody against Epstein-Barr virus viral capsid antigen; FTA-ABS: fluorescent treponemal antibody absorption test; Herpes I IgM: immunoglobulin M antibody against herpes simplex virus type 1; Herpes II IgM: immunoglobulin M antibody against herpes simplex virus type 2

Test	Result	Reference Range
Chamydia IgA	<5.0	<5.0 index – Negative
Cytomegalovirus IgM II	<18	< 18 U/mL – Negative 18–22 U/mL – Undetermined
Epstein-Barr-VCA IgM	<20	<20 U/mL – Negative 20–40 U/mL – Undetermined
FTA-ABS	Non reactive	Non reactive
Herpes I IgM	<0.9	<0.9 – Negative 0.9–1.1 – Undetermined
Herpes II IgM	<0.9	<0.9 – Negative 0.9–1.1 – Undetermined

Based on the combination of rash morphology, sedentary behavior, psychological stressors, and overall clinical findings, a diagnosis of presumed cutaneous small-vessel vasculitis, likely stress-induced, was made.

The patient was started on oral deflazacort, 30 mg twice daily for the first three days to achieve rapid inflammation control. From Day four, the dose was reduced to 30 mg once daily for three days, followed by a taper: 20 mg on Day seven, 15 mg on Day eight, 10 mg on Day nine, and 5 mg on Day 10, at which point the corticosteroid was discontinued. Topical treatment included mometasone furoate 0.1% cream, applied twice daily for two weeks. Adjunctive skin care consisted of a relipidizing cream containing Avène Thermal Spring Water applied daily and a gentle emollient cleansing gel with Rhealba® Oat Plantlet Extract to support barrier integrity and relieve pruritus. The regimen was well tolerated, with significant improvement in symptoms and no reported adverse effects. The lesions resolved over the following two weeks without scarring.

The patient was referred for both psychiatric and psychological therapy. In addition, stress management strategies were discussed, including mindfulness-based techniques, breathing exercises, and cognitive-behavioral strategies aimed at reducing anxiety and promoting emotional regulation. However, the patient faced significant economic limitations that made it difficult to access ongoing mental health services. Furthermore, support options for low-income individuals in the region were limited, which further restricted access to psychological and psychiatric care. 

## Discussion

Urticarial vasculitis (UV) is a rare form of small-vessel vasculitis that may present with both cutaneous and systemic symptoms [4,5]. UV is divided into two subtypes based on complement levels: normocomplementemic urticarial vasculitis (NUV) and hypocomplementemic urticarial vasculitis (HUV). NUV is more common and typically limited to the skin [6]. In this case, the patient had normal complement levels and evidence of systemic involvement, consistent with a diagnosis of NUV. 

Clinically, UV can be confused with chronic spontaneous urticaria (CSU); however, unlike CSU, UV lesions tend to persist longer than 24 hours, may cause a burning or painful sensation, and often leave residual hyperpigmentation. Additionally, systemic symptoms such as fatigue, arthralgia, and gastrointestinal disturbances are more suggestive of UV [3]. Our patient’s presentation, marked by persistent urticarial lesions, abdominal pain, diarrhea, arthralgia, myalgia and skin tenderness, was consistent with UV rather than CSU. The core differences between HUV, NUV and CSU are described in Table 4.

**Table 4:** Core differences between HUV, NUV and CSU This table was created independently by the authors using information obtained from references [3-6]. NUV: normocomplementemic urticarial vasculitis; HUV: hypocomplementemic urticarial vasculitis; CSU: chronic spontaneous urticaria.

Feature	NUV	HUV	CSU
Complement Levels	Normal	Low	Not applicable
Frequency	More common	Less common	Most common chronic urticaria
Systemic Involvement	Typically limited to skin; systemic symptoms less common	More common systemic symptoms	Usually no systemic symptoms
Lesion Duration	Persist >24 hours	Persist >24 hours	Typically last <24 hours
Lesion Sensation	Burning or painful sensation	Burning or painful sensation	Usually itchy, not painful or burning
Residual Skin Changes	Often hyperpigmentation	Often hyperpigmentation	Usually no residual pigmentation
Associated Symptoms	Usually absent	Fatigue, arthralgia, GI symptoms	No systemic symptoms
Treatment	NSAIDs, Antihistamines, corticosteroids, immunomodulators if needed	Same, corticosteroids especially important	Antihistamines mainstay, corticosteroids if refractory
Diagnosis	Clinical presentation, normal complement, skin biopsy confirms vasculitis	Clinical presentation, low complement, biopsy confirms vasculitis	Clinical diagnosis, no vasculitis on biopsy

The diagnostic process includes taking a medical history, performing a physical examination, and conducting laboratory tests and imaging studies. A skin biopsy can help confirm the diagnosis [7]. The pathophysiology is mediated by type III hypersensitivity, in which antigen-antibody complexes deposit in small blood vessels, activating the complement cascade and promoting further inflammation and vascular damage. Systemic symptoms are more common in HUV than in NUV [8].

This case highlights a rare presentation of NUV with systemic symptoms in a young adult, with the illness onset temporally associated with psychological stress and comorbid untreated GAD. While UV is often idiopathic or linked to autoimmune or drug-related triggers, stress is an underreported but plausible factor. There has been a reported association between stress, childhood trauma, and dermatologic disease, which has been closely related to anxiety [9].

Treatment of UV is based on severity and systemic involvement. While antihistamines can provide symptom relief, corticosteroids are effective in managing over 80% of cases and remain the cornerstone of treatment [2,7]. Other immunomodulatory agents, including hydroxychloroquine, colchicine, dapsone, and biologics such as omalizumab or rituximab, may be employed in refractory or systemic cases [2]. In our case, the patient responded well to corticosteroid therapy, with full resolution of symptoms and no recurrence during follow-up.

This case underscores the importance of considering urticarial vasculitis (UV) in the differential diagnosis of persistent urticarial lesions, particularly in patients presenting with systemic symptoms or poor response to antihistamines. It also highlights the potential role of psychological stress as a triggering factor for UV, especially in individuals with underlying psychiatric conditions. These findings emphasize the necessity of a comprehensive biopsychosocial approach to the evaluation and management of dermatologic diseases, including timely referral for psychological and psychiatric care.

## Conclusions

Urticarial vasculitis remains a diagnostic and therapeutic challenge due to its heterogeneous presentation and often idiopathic etiology. This case illustrates a rare instance of normocomplementemic urticarial vasculitis (NUV) likely triggered by psychological stress in a patient with untreated generalized anxiety disorder (GAD). Clinicians should maintain a high index of suspicion for UV in patients presenting with persistent urticarial lesions and systemic symptoms. A comprehensive approach that includes psychological and psychiatric assessment and treatment, alongside corticosteroid therapy, may be beneficial in such cases. Further research is warranted to elucidate the relationship between emotional stress and immune-mediated vasculitides.
